# Cardiomyocyte electrical-mechanical synchronized model for high-content, dose-quantitative and time-dependent drug assessment

**DOI:** 10.1038/s41378-021-00247-0

**Published:** 2021-03-25

**Authors:** Jiaru Fang, Xinwei Wei, Hongbo Li, Ning Hu, Xingxing Liu, Dongxin Xu, Tao Zhang, Hao Wan, Ping Wang, Xi Xie

**Affiliations:** 1 The First Affiliated Hospital of Sun Yat-Sen University; School of Electronics and Information Technology, Guangdong Province Key Laboratory of Display Material and Technology, Sun Yat-sen University, Guangzhou, 510006 China; 2 Biosensor National Special Laboratory, Key Laboratory of Biomedical Engineering of Ministry of Education, Department of Biomedical Engineering, Zhejiang University, Hangzhou, 310027 China; 3 State Key Laboratory of Transducer Technology, Chinese Academy of Sciences, Shanghai, 200050 China; 4 School of Biomedical Engineering, Sun Yat-sen University, Guangzhou, 510006 China

**Keywords:** Electrical and electronic engineering, Biosensors, Biosensors

## Abstract

Cardiovascular diseases have emerged as a significant threat to human health. However, drug development is a time-consuming and costly process, and few drugs pass the preclinical assessment of safety and efficacy. The existing patch-clamp, Ca^2+^ imaging, and microelectrode array technologies in cardiomyocyte models for drug preclinical screening have suffered from issues of low throughput, limited long-term assessment, or inability to synchronously and correlatively analyze electrical and mechanical signals. Here, we develop a high-content, dose-quantitative and time-dependent drug assessment platform based on an electrical-mechanical synchronized (EMS) biosensing system. This microfabricated EMS can record both firing potential (FP) and mechanical beating (MB) signals from cardiomyocytes and extract a variety of characteristic parameters from these two signals (FP–MB) for further analysis. This system was applied to test typical ion channel drugs (lidocaine and isradipine), and the dynamic responses of cardiomyocytes to the tested drugs were recorded and analyzed. The high-throughput characteristics of the system can facilitate simultaneous experiments on a large number of samples. Furthermore, a database of various cardiac drugs can be established by heat map analysis for rapid and effective screening of drugs. The EMS biosensing system is highly promising as a powerful tool for the preclinical development of new medicines.

## Introduction

With changes in human diets and lifestyles in modern society, various diseases have emerged as significant threats to human health. At present, cardiovascular diseases are gradually increasing in incidence^1,2^ and have become the leading cause of death among adults, accounting for ~40% of annual mortality^3,4^. Numerous medicines have been developed and applied in order to effectively prevent and treat cardiovascular diseases^5^. However, drug development is a costly and time-consuming process with a low success rate and a high withdrawal risk. Therefore, it is necessary to carry out comprehensive preclinical research on the efficacy and safety of drugs to avoid potential threats to human health as well as immense economic losses^6–8^.

Cultured cardiomyocytes are a conventional in vitro model for preclinical analysis of drugs, typically by cell-based biosensing detection technologies, which include invasive/label-based detection technologies and noninvasive/label-free detection technologies^9–13^. For example, patch-clamp recording^14,15^ and Ca^2+^ imaging^16,17^ are typical invasive detection techniques for a cardiomyocyte model. The invasive technology of patch-clamp recording^18,19^ can monitor the activity of ion channels in the cell membrane, providing a high-quality signal that serves as the gold standard, but this technique suffers from low throughput due to its complicated operating procedure. On the other hand, label-based Ca^2+^ fluorescence imaging can reflect the mechanical activity of cardiomyocytes^20^, but it is challenging to apply to long-term dynamic assessment due to invasiveness and phototoxicity. In order to overcome these drawbacks, noninvasive and label-free biosensing technologies have been developed, such as microelectrode array (MEA) and electrical cell-based impedance sensing (ECIS) technology^21–25^. MEA can record the electrical signals of cultured cardiomyocytes over long periods in a high-throughput, dynamic manner^26^, while ECIS can monitor the mechanical beating of cardiomyocytes. However, these technologies can monitor only a single parameter—either electrical or mechanical signals. The inability to record electrical and mechanical signals simultaneously has hindered the acquisition of synchronous and correlative information between the electrical and mechanical properties of cardiomyocytes. Currently, commercial instruments such as MEA2100-Systems (Smart Ephys)^27,28^ and CardioExcyte 96 (Nanion)^29,30^ are well-established platforms for in vitro pharmaceutical screening and are generally applied solely to record electrophysiological signals from cardiomyocytes. While CardioExcyte 96 can record both electrophysiological signals and mechanical beating signals in separate modes, synchronous electromechanical integrated signal recording by simultaneous recording of both electrophysiological and mechanical signals has not been realized. Consequently, it is necessary to develop a noninvasive, label-free and multiparameter platform for preclinical drug assessment.

**Fig. 1:**
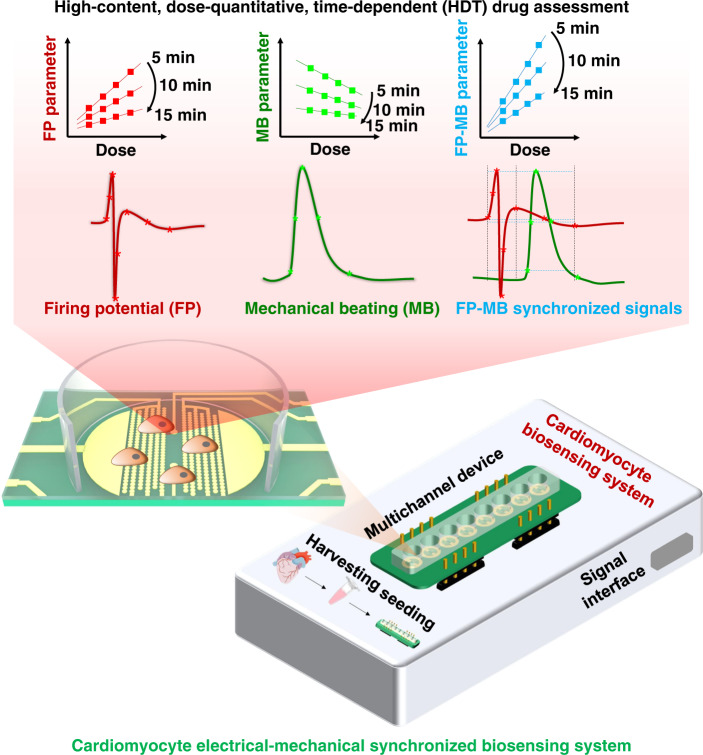
Schematic diagram of high content, dose-quantitative and time-dependent drug assessment of cardiomyocytes by the EMS biosensing system. The high-throughput cardiomyocyte-based biosensing system consists of a multichannel integrated device with an electrical-mechanical synchronized recording module. The firing potential (FP) and mechanical beating (MB) signals can be simultaneously collected by the microelectrodes and interdigitated electrodes on the device. By extracting the features of the FP, MB, and FP–MB synchronized signals, it is possible to analyze the effects of drugs on cardiomyocytes in a high-content, dose-quantitative and time-dependent manner

Here, we develop a high-content, dose-quantitative and time-dependent drug assessment platform for investigating the effects of cardiac drugs; this system can simultaneously monitor both electrical and mechanical signals from cardiomyocytes (Fig. 1). This electrical-mechanical synchronized (EMS) biosensing system can record both firing potential (FP) and mechanical beating (MB) signals from cardiomyocytes and extract a variety of characteristic parameters from these two signals (FP–MB) for further analysis. This system was applied to test typical ion channel drugs (lidocaine and isradipine), and the feature parameters of FP, MB and FP–MB were derived for quantitative analysis of drugs. Moreover, the dynamic responses of cardiomyocytes to the tested drugs were recorded and analyzed to assess the time-dependent effects of the drugs. In addition, the high-throughput characteristics of the system facilitated simultaneous experiments on a large number of samples. With this system, a database of various cardiac drugs can be established by heat map analysis for rapid and effective screening of drugs. The EMS biosensing system can benefit high-content, dose-quantitative and time-dependent drug assessment and will serve as a promising and powerful tool for the preclinical development of new medicines.

## Experimental methods

### Device fabrication

**Fig. 2:**
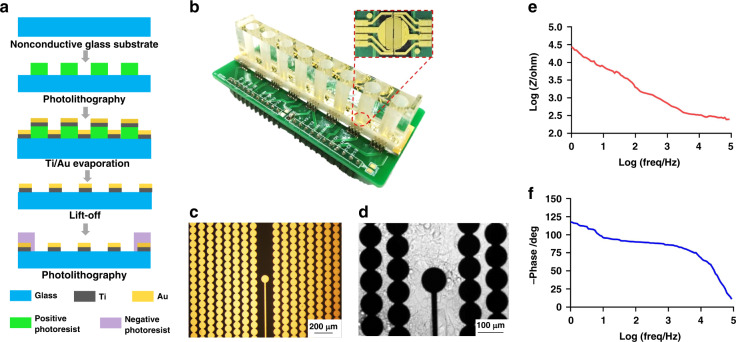
Characterization of the EMS biosensing system. (**a**) Image of integrated device. The integrated device has eight devices that are mounted on a nonconductive glass substrate. (**b**) Microscope image of the integrated device. The microscopic view shows that each device in the integrated device contains microelectrodes, reference electrodes and interdigitated electrodes. (**c**) The fabrication procedures for integrated devices with electrodes. (**d**) Microscope image of cells cultured on the device. (**e**) The impedance diagram of the integrated device. The device was tested with an electrochemical impedance sweep spectrum; when the excitation frequency was 1 Hz to 1000 Hz, the integrated device impedance was large, and when the frequency was greater than 1000 Hz, the impedance was small. (**f**) Phase diagram of the electrical impedance of the integrated device. When the excitation frequency is less than 1 kHz, the phase angle of the signal measured by electrical impedance is approximately −120° to −80°, while the phase angle decreases as the frequency increases

The microelectrode-integrated device consists of a total of 8 independent devices, where each independent device contains a microfabricated electrode array, reference electrodes, and interdigitated electrodes mounted on a nonconductive glass substrate. The fabrication of the microelectrodes is based on standard photolithography lift-off processes. Briefly, the electrode was first patterned by UV photolithography and thermal evaporation with 20 nm Ti/100 nm Au. The leads of the electrodes were insulated with the negative photoresist SU-8 2002 by photolithography (Fig. 2a). Generally, the size of each cell is 10–30 µm, and the diameter of each microelectrode is approximately 10–100 µm, which makes them appropriately sized for single cells. A smaller electrode area will increase the impedance of the electrode and the noise, while a larger electrode area will reduce the impedance of the electrode and lead to an increase in the leakage current and a decrease in the signal amplitude. When the electrode array is in a shared solution environment, there will be interference between the electric fields of the electrodes; accordingly, the pitch of the electrodes should not be too small. In order to ensure that no single cell would cross two electrodes, the interdigitated electrodes were designed with a diameter of 90 µm and a distance of 120 µm between neighboring branches, while the two microelectrodes were designed with a pitch of 3 mm in the middle and a diameter of 100 µm (Fig. 2b–d). Finally, the device was assembled into a polymethyl methacrylate (PMMA) chamber for cell culture.

### Characterization of the integrated device

Electrochemical impedance spectroscopy was used to determine the electrochemical characteristics of the integrated device. AC impedance spectroscopy was performed with an electrochemical workstation two-electrode system using frequency sweeps of 1 Hz to 10 kHz. The electrical impedance values and phase results are shown in Fig. 2e, f. When the value of the excitation frequency is in a low-frequency range of 1 Hz to 1000 Hz, the integrated device impedance Z is large, ranging from 10^3^ to 10^5^ Ω. At frequencies greater than 1000 Hz, however, the integrated device impedance Z is small. The phase information of the electrical impedance value supports a similar conclusion. When the excitation frequency is in a low-frequency band (less than 1 kHz), the phase angle of the signal measured by the electrical impedance is in the vicinity of −120° to −80°, and the phase angle gradually decreases in the subsequent frequency interval. The low-frequency phase value indicates the process of capacitor charging taking place on the electrode surface of what is called a double-layer capacitor. For the cleaning protocol, the integrated device was soaked in 1% cleaning solution (Alconox, USA), placed in a refrigerator at 4 °C overnight, cleaned with distilled water five times, and dried for storage. The integrated device can be reused at least five times.

### Cell culture

Devices were sterilized with 75% ethanol under UV exposure for 2 h in a biosafety cabinet, and the cleaned devices were coated with a 10 mg/ml fibrin solution in a 4 °C refrigerator overnight to enhance cell adhesion to the gold surface. Human induced pluripotent stem cell-derived cardiomyocytes (hiPSC-CMs), specifically Cor.4U from Ncardia, were thawed and cultured in antibiotic-free medium (Ncardia) for two days before seeding. The culture conditions for the cells were optimized before the bioassay. As shown in Supplementary Fig. 3, the recording of cultured cardiomyocytes (e.g., firing rate, firing amplitude, beating rate, and beating amplitude) was optimized by investigating 3 consecutive days of synchronized signal data, where the cells were cultured at different densities of 2.0 × 10^5^, 3.0 × 10^5^, and 4.0 × 10^5^ cells/mL. The cells are generally considered to be in the optimized condition for recording when they achieve a normal beating rate (~60 times/min) and the maximum amplitude of firing potential. According to the statistical results, cells at a density of 3.0 × 10^5^ cells/mL exhibited the best signal for 2 consecutive days of culture. For example, the firing rate and amplitude increased to ~60 times/min and 428 ± 9.6 μV, respectively, on the second day. In addition, the firing amplitude of cardiomyocytes was larger at a culture density of 3.0 × 10^5^ cells/ml than at other densities (2.0 × 10^5^ cells/ml produced 215 ± 6.3 μV, and 4.0 × 10^5^ cells/ml produced 263 ± 11.1 μV). Therefore, day 2 was selected as the optimal experimental time point. Cells were transferred into the device at a density of 3.0 × 10^5^ cells/ml, and the medium was exchanged every two days to maintain sufficient nutrients.

### Working principle of the cardiomyocyte EMS biosensing system

An EMS biosensing system consists of an integrated microelectrode device and a synchronized recording system. The integrated device monitors the electrical and mechanical signals of hiPSC-CMs with microelectrodes and interdigitated electrodes. The cardiomyocytes are cultured on electrodes for two days, and the electrical signals are recorded by a microelectrode, while the impedance fluctuations induced by rhythmic mechanical beating are recorded by the interdigitated electrodes. In order to achieve synchronous recording of FP and MB signals, a 3 kHz low-pass filter is applied to filter the high-frequency AC drive signal of the interdigitated electrodes from the FP signal. The optimized FP signals are sampled by an analog-to-digital converter (ADC) on a data acquisition module. MB signals are recorded based on the impedance variation of the interdigitated electrode at a given working frequency (10 kHz). The generated AC current signals are converted to an AC voltage signal by a transimpedance amplifier (TIA). The voltage signal is conditioned by a high-pass amplification module and sampled, and the fast Fourier transform (FFT) is performed by the Data acquisition (DAQ) module. Thus, the recording system collects signal data from different devices at the same time and records both firing potentials and mechanical signals from cardiomyocytes synchronously from the separate devices.

### Drug assay

Cardiomyocyte status was initially examined every 1 h after seeding. Drug assays are conventionally performed when cardiomyocytes present stable electrical and mechanical signals after culturing for days. Before drug treatment, the culture medium was exchanged with fresh medium. After 4 h of recovery, the stable baseline electrical-mechanical signals of cardiomyocytes were first recorded by a synchronized recording system; different concentrations of lidocaine or isradipine were then administered to the cells, and signals were recorded every 5 min. In order to improve solubility, the drugs were dissolved in dimethyl sulfoxide (DMSO); the final concentration of DMSO in the cell culture medium was 0.2% Fibrin, l-glutamine, fetal bovine serum (FBS), isradipine, lidocaine, and DMSO were purchased from Sigma–Aldrich.

### Signal processing

Subsequent signal processing and feature extraction were carried out in a customized LabVIEW program (National Instrument, USA). In order to maintain the high fidelity of the FP and MB signals, the noise was removed, and feature points were extracted from the signals by an adaptive threshold algorithm^25,31–34^. E1–E8 are defined as the feature points of FP signals: 10% of the rising edge (E1), 50% of the rising edge (E2), the peak point (E3), the valley point (E4), 50% of the second rising edge (E5), the second peak point (E6), 50% of the second falling edge (E7), and the second valley point (E8). Meanwhile, M1–M5 are defined as the feature points of MB signals: 10% of the rising edge (M1), 50% of the rising edge (M2), the peak point (M3), 50% of the falling edge (M4), and 90% of the falling edge (M5). Moreover, electrical-mechanical correlation information was derived between the E and M feature points.

### Statistical analysis

Signal processing was performed by a customized LabVIEW program. All results and error bars are expressed as the mean±standard deviation (SD). The statistical analysis was performed using Prism 8.0 (GraphPad, USA) or Excel 2016 (Microsoft, USA).

## Results and discussion

### Establishment and characterization of the cardiomyocyte EMS model

**Fig. 3:**
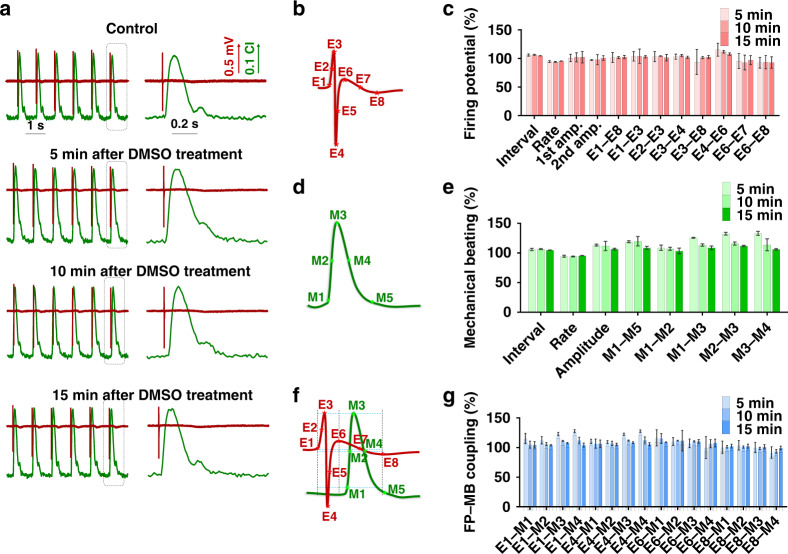
Characterization of EMS model. **a** Typical EMS signals before and after 2% DMSO treatment. Human induced pluripotent stem cell-derived cardiomyocytes (iPSC-CMs) present mature and stable EMS signals after four days of culture. The right panel is a close-up view of the signal in the dashed box of the left panel. **b** Schematic of eight feature points (E1–E8) of the extracellular potential signal. Feature points E1–E8 are 10% of the rising edge (E1), 50% of the rising edge (E2), the peak (E3), the valley (E4), 50% of the second rising edge (E5), the second peak (E6), 50% of the second falling edge (E7), and the second valley (E8). **c** Stability analysis of each feature parameter of extracellular potential signals under DMSO treatment for 5–15 min, including the firing interval, firing rate, first amplitude, second amplitude, and durations between feature points (*n* = 4). **d** Schematic of the five feature points (M1–M5) of mechanical beating signals. M1–M5 are 10% of the rising edge, 50% of the rising edge, the peak, 50% of the falling edge, and 90% of the falling edge, respectively. **e** Stability analysis of each feature parameter of mechanical beating signals under DMSO treatment for 5–15 min, including beating interval, beating rate, beating amplitude and durations between feature points (*n* = 4). **f** Schematic of the E-M duration features of the EMS signals. **g** Stability analysis of feature parameters of EMS signals under 5–15 min of DMSO treatment (*n* = 4)

In order to maintain human-specific characteristics, hiPSC-CMs were selected as the cells with which to establish the electrical-mechanical synchronized model. The integrated device cultured the optimal density of cardiomyocytes to ensure long-term culture with high-quality electrical-mechanical signals. After 2 days of culture, synchronous FP and MB signals were present and could be recorded as stable electrical-mechanical signals. In order to establish the cardiomyocyte EMS model for drug assessment, the performance of the model was first characterized based on the common solvent DMSO. As shown in Fig. 3a, the short-term (5, 10 and 15 min) stability of the high-content cardiomyocyte synchronized model was verified under DMSO solvent treatment. Compared with the control condition, the FP (red) and MB (green) signals displayed similar profiles under 15 min of DMSO treatment.

In order to establish a high-content model, the feature points were extracted from the electrical-mechanical signals using LabVIEW. In total, 8 feature points (E1–E8) were defined on the FP signal, as shown in Fig. 3b (E1: 10% of the rising edge; E2: 50% of the rising edge; E3: the peak point; E4: the valley point; E5: 50% of the second rising edge; E6: the second peak point; E7: 50% of the second falling edge; and E8: the second valley point). In total, 12 FP parameters, including firing interval; firing rate; first amplitude; second amplitude; and the durations of E1–E8, E1–E3, E2–E3, E3–E4, E4–E8, E4–E6, E6–E7 and E6–E8, were extracted based on these feature points of FP signals, and the maximum fluctuation was below 8.2% within 15 min of 0.2% DMSO treatment (Fig. 3c)^35^. Meanwhile, 5 feature points (M1–M5) were defined on the MB signal, as shown in Fig. 3d (M1: 10% of rising edge, M2: 50% of rising edge, M3: peak point, M4: 50% of the falling edge, and M5: 90% of the falling edge). Eight MB parameters, including beating interval; beating rate; peak amplitude; and the durations of M1–M5, M1–M2, M1–M3, M2–M3 and M3–M4, were extracted based on these feature points of MB signals, and the maximum fluctuation was below 14.6% within 15 min of DMSO treatment (Fig. 3e). Moreover, based on the feature points of FP and MB, a total of 16 electrical-mechanical correlation parameters, namely, the durations of E1–M1, E1–M2, E1–M3, E1–M4, E4–M1, E4–M2, E4–M3, E4–M4, E6–M1, E6–M2, E6–M3, E6–M4, E8–M1, E8–M2, E8–M3 and E8–M4, were extracted, as shown in Fig. 3f, and the maximum fluctuation was below 12.1% within 15 min of DMSO treatment (Fig. 3g). According to the statistical analysis, 12 electrical parameters, 8 mechanical parameters, and 16 electrical-mechanical parameters were initially selected to establish a reliable high-content cardiomyocyte EMS model. This model uses hiPSC-CMS as a sensitive element and combines it with the intrinsic physiological properties of electroexcitation-mechanical coupling of cardiomyocytes, which can be applied for subsequent drug assessment.

### Dose-quantitative assessment of ion channel drugs

**Fig. 4:**
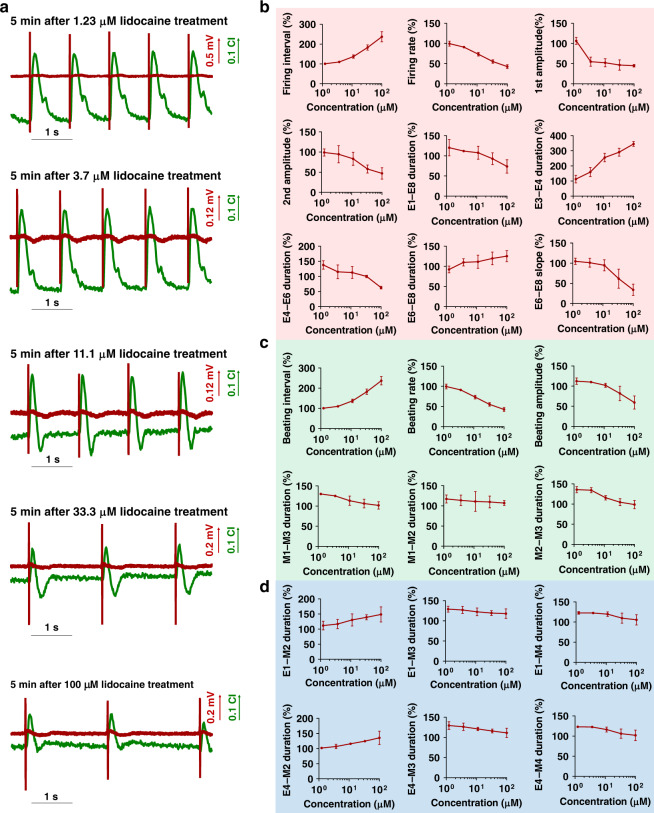
Assessment of an Na^+^ channel drug (lidocaine) by the EMS model. **a** Typical extracellular potential and mechanical beating signals of cardiomyocytes under lidocaine treatment at different concentrations (1.23 μM, 3.7 μM, 11.1 μM, 33.3 μM, 100 μM). **b** Statistical analysis of feature parameters of extracellular potential signals under lidocaine treatment at five different concentrations. **c** Statistical analysis of the feature parameters of mechanical beating signals under lidocaine treatment at five different concentrations. **d** Statistical analysis of feature parameter EMS signals under lidocaine treatment at five different concentrations (*n* = 4)

Electrophysiology can effectively reflect the functions of ion channels. The inflow and outflow of Na^+^, Ca^2+^, and K^+^ can evoke action potentials and cascades of mechanical contraction. In addition, these ion channels are important target sites for drug treatment; thus, the quantitative assessment of ion channel drugs plays a significant role in drug screening. In order to determine the dose-quantitative function of EMS in a cardiomyocyte model, typical ion channel blockers (lidocaine and isradipine) were employed to test the performance of the cardiomyocyte synchronization model. Figure 4a presents typical EMS signals of cardiomyocytes under 5 min lidocaine (a Na^+^ channel blocker that inhibits, signal transmission, and contractility in cardiomyocytes^36–38^) treatment with different drug concentrations (1.23 µM, 3.7 µM, 11.1 µM, 33.3 µM, and 100 µM). Lidocaine markedly inhibits FP and MB signals by reducing the FP amplitude, firing rate, MB amplitude, and beating rate. With increasing drug concentrations, the firing rate and beating rate of cardiomyocytes significantly decreased by 57.58%. In order to explore drug efficacy in a high-content level of detail, the EMS signals were further analyzed based on the FP, MB and FP–MB feature parameters. In total, 9 FP feature parameters (firing interval, firing rate, first amplitude, second amplitude, E1–E8 duration, E3–E4 duration, E4–E6 duration, E6–E8 duration and E6–E8 slope) showed dose-dependent responses to lidocaine (Fig. 4b), and the firing rate, E3–E4 duration and E6–E8 duration increased, while other parameters decreased with different degrees of sensitivity. Meanwhile, 6 MB feature parameters (beating interval, beating rate, beating amplitude, M1–M2 duration, M1–M3 duration and M2–M3 duration) showed dose-dependent responses (Fig. 4c), and the statistical results concerning the firing interval, beating interval, firing rate and beating rate were similar due to excitation–contraction coupling.

Moreover, since the electrical and mechanical signals of cardiomyocytes are well correlated, FP–MB correlative feature parameters also have potential for drug assessment. From the statistical analysis shown in Fig. 4d, 6 FP–MB parameters (E1–M2 duration, E1–M3 duration, E1–M4 duration, E4–M2 duration, E4–M3 duration and E4–M4 duration) showed detectable dose-dependent responses. The E1–M2 duration and E4–M2 duration were prolonged by 26.49% and 33.66%, respectively, while the E1–M3 duration, E1–M4 duration, E4–M3 duration and E4–M4 duration were shortened by 9.30%, 17.21%, 13.95% and 17.89%, respectively. When compared with the isolated parameters from FP or MB signals, the correlative parameters from FP–EB synchronized signals can provide new indexes for drug assessment. The results above demonstrate that the FP, MB and FP–MB feature parameters can provide high-content and dose-quantitative analysis for drug assessment.

**Fig. 5:**
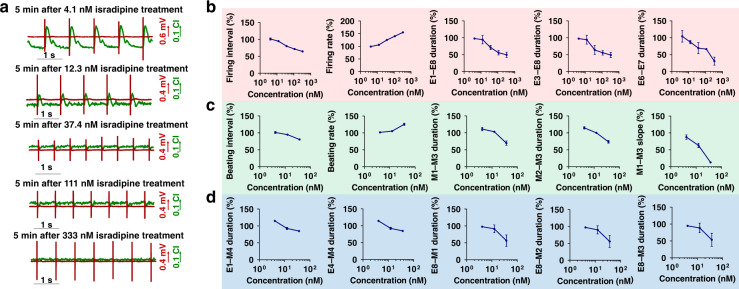
Assessment of a Ca^2+^ channel drug (isradipine) with the EMS model. **a** Typical extracellular potential and mechanical beating signals of cardiomyocytes under isradipine treatment at different concentrations (4.1 nM, 12.3 nM, 37.4 nM, 111 nM, 333 nM). **b** Statistical analysis of five feature parameters of extracellular potential signals under isradipine treatment at five different concentrations. **c** Statistical analysis of the feature parameters of mechanical beating under isradipine treatment at concentrations of 4.1 nM, 12.3 nM and 37.4 nM. **d** Statistical analysis of feature parameter EMS signals under isradipine treatment at concentrations of 4.1 nM, 12.3 nM and 37.4 nM (*n* = 4)

Ca^2+^ channels play a significant role in action potentials, especially in triggering the mechanism of excitation–contraction coupling; thus, isradipine (a Ca^2+^ channel blocker that inhibits Ca^2+^ channels and shortens the action potential of cardiomyocytes^39^) was administered to test the EMS model. Figure 5a presents typical EMS signals of cardiomyocytes under 5 min of isradipine treatment at different concentrations (4.1 nM, 12.3 nM, 37.4 nM, 111 nM, 333 nM); the rates of FP and MB signals were obviously accelerated. EMS signals were further analyzed based on the FP, MB, and FP–MB feature parameters. In total, 5 FP feature parameters (firing interval, firing rate, E1–E8 duration, E3–E8 duration and E6–E7 duration) showed dose-dependent responses to isradipine (Fig. 5b). As shown by the statistical analysis, the firing rate of FP increased, while other parameters declined. However, the mechanical beating of cardiomyocytes was strongly inhibited under isradipine treatment at high concentrations (111 nM and 333 nM); thus, the feature parameters of MB and FP–MB signals were further analyzed with a focus on lower concentrations. MB and FP–MB signals showed dose-dependent responses to isradipine ranging from 4.1 nM to 37.4 nM (Fig. 5c, d). In total, 5 MB feature parameters (beating interval, beating rate, M1–M2 duration, M1–M3 duration, and M2–M3 duration) were analyzed. The beating interval and beating rate had similar results to the firing interval and firing rate, respectively, due to excitation–contraction coupling. The E1–E8 duration, E3–E8 duration, and E6–E7 duration declined by 49.19%, 49.63% and 70.54%, respectively. As shown in Fig. 4d, 5 FP–MB feature parameters (E1–M4 duration, E4–M4 duration, E8–M1 duration, E8–M2 duration, and E8–M3 duration) decreased with different degrees of sensitivity. These results demonstrate the feasibility of high-content and dose-quantitative drug evaluation with FP, MB and FP–MB feature parameters.

### Time-dependent assessment of ion channel drugs

**Fig. 6:**
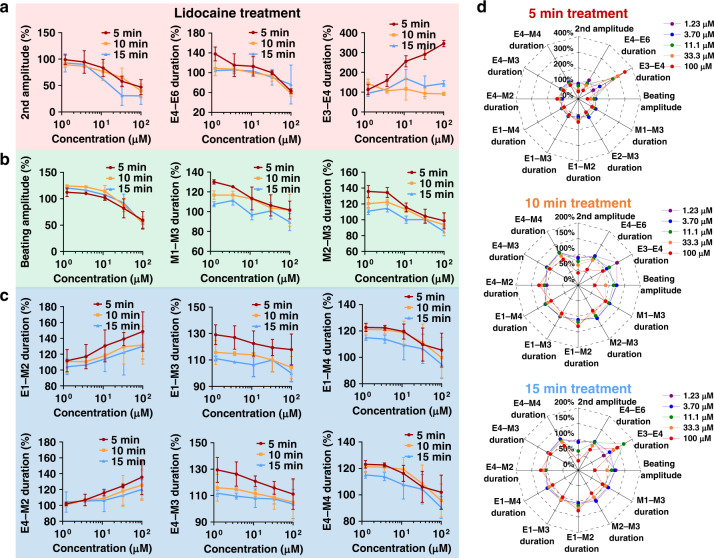
Time-dependent assessment of lidocaine by the EMS model. **a** Statistical analysis of the time-dependent parameters under lidocaine treatment, including the second amplitude, E4–E6 duration and E3–E4 duration of extracellular potential signals. **b** Statistical analysis of the time-dependent parameters under lidocaine treatment, including the beating amplitude, M1–M3 duration and M2–M3 duration of mechanical beating signals. **c** Statistical analysis of the time-dependent parameters under lidocaine treatment, including the E1–M2 duration, E1–M3 duration, E1–M4 duration, E4–M2 duration, E4–M3 duration and E4–M4 duration. **d** Radar charts of all feature parameters analyzed in **a**–**c** after 5, 10 and 15 min of drug treatment. In the radar charts, different concentrations of lidocaine (1.23 μM, 3.7 μM, 11.1 μM, 33.3 μM, 100 μM) displayed specific patterns for a given duration of treatment

In addition to dose-quantitative assessment, time-dependent assessment also provides an important reflection of drug efficacy. In order to gauge the time-dependent assessment function of the EMS model, the dynamic feature parameters were extracted and analyzed from the EMS signals. The feature parameters of cardiomyocytes were investigated within 15 min due to the rapid efficacy of the drug on cardiomyocytes. Compared with the dose-dependent assessment, 3 FP feature parameters (2nd amplitude, E4–E6 duration, and E3–E4 duration), 3 MB feature parameters (beating amplitude, M1–M3 duration and M2–M3 duration) and 6 FP–MB feature parameters (E1–M2 duration, E1–M3 duration, E1–M4 duration, E4–M2 duration, E4–M3 duration and E4–M4 duration) presented time-dependent responses to lidocaine. As shown in Fig. 6a–c, these 12 feature parameters changed within 15 min of treatment, which simultaneously indicated the efficacy of the drug in the dimensions of dose and time dimensions. In order to intuitively display the dose-quantitative and time-dependent efficacy of the drug in terms of these 12 parameters, a radar chart was plotted, as shown in Fig. 6d. The specific response of the model to lidocaine showed obvious changes in various parameters. In addition, the radar map integrated the parameters into a diagram for comparison, which provided an intuitive analysis method to reflect the efficacy of the drug in the dimensions of dose and time.

A time-dependent assessment of isradipine was also performed with the EMS model. In contrast to lidocaine, only 4 feature parameters of isradipine (firing interval, firing rate, E6–E7 duration and M1–M3 duration) presented time-dependent responses within 15 min of treatment (Supplementary Fig. S1a), but these parameters still showed the efficacy of the drug in the dimensions of dose and time. Each specific radar map clearly showed the changes in feature parameters, which reflect the specific response of the model to isradipine. Compared with the conventional parameters, the high-content feature parameters of the EMS model increased the opportunity and capacity to achieve both dose-quantitative and time-dependent analysis. The results above demonstrated that the high-content EMS signals provided additional potential feature parameters for ion channel assessment in a dose-quantitative and time-dependent manner.

### Visualized analysis of EMS model

**Fig. 7:**
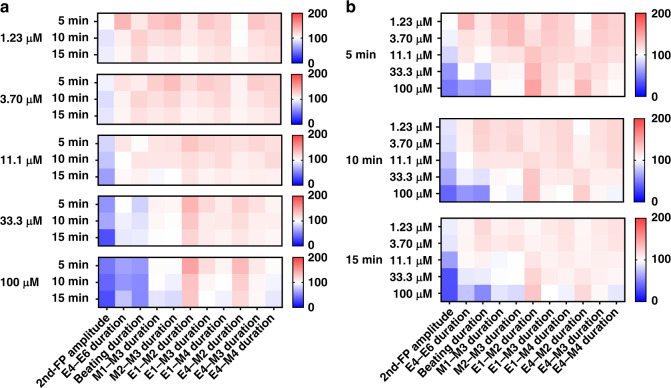
Visualized analysis of the EMS model under lidocaine treatment using a heat map. **a** Dose-quantitative specific patterns in response to 5, 10 and 15 min of lidocaine treatment. Heat maps with the concentrations ranging from 1.23 to 100 μM treatment at each time presented similar patterns, indicating similar variations in the feature parameters. **b** Time-dependent specific patterns over 15 min of lidocaine treatment at doses of 1.23 μM, 3.7 μM, 11.1 μM, 33.3 μM, and 100 μM. Heat maps reflecting 5–15 min of treatment with each dose also presented similar and obvious changes in feature parameters

In order to visually display the feature parameters of the EMS model, heat maps were introduced to simultaneously present the dose-quantitative and time-dependent responses to the drugs. The specific patterns of the EMS model are shown on the heat map (Fig. 7). In total, 2 FP feature parameters (2nd amplitude and E4–E6 duration), 3 MB feature parameters (beating amplitude, M1–M3 duration, and M2–M3 duration), and 6 FP–MB feature parameters (E1–M2 duration, E1–M3 duration, E1–M4 duration, E4–M2 duration, E4–M3 duration, and E4–M4 duration) presented the visualized dose-quantitative and time-dependent changes under lidocaine treatment. Figure 7a shows a dose-quantitative heat map reflecting 5, 10 and 15 min of lidocaine treatment. Heat maps for lidocaine treatment presented similar patterns from 1.23 to 100 μM, and each feature parameter displayed dose-quantitative changes. In order to further analyze the time dependence of drug assessment, the heat maps were converted to the time-dependent response to each dose over the course of 15 min (Fig. 7b). On each concentration map, the feature parameters exhibited obvious variation over 15 min of lidocaine treatment. All parameters showed time-dependent changes at each concentration, among which the beating amplitude displayed a slight increasing trend over time and the other parameters all decreased. The results obtained from the heat map were consistent with the statistical analysis above.

Similarly, the dose-quantitative and time-dependent responses to isradipine were visually analyzed using heat maps (Supplementary Fig. S2). In total, 4 feature parameters of FP and MB signals (firing interval, firing rate, E6–E7 duration, and M1–M3 duration) presented visualized dose-quantitative and time-dependent responses under isradipine treatment. A total of 3 parameters of the FP signal possessed specific patterns, as shown in Supplementary Fig. S2a. The changes in firing interval, firing rate, and E6–E7 duration with drug concentration showed a specific pattern where the firing rate increased with concentration, while the firing interval and E6–E7 duration decreased in a dose-dependent manner. Notably, M1–M3 disappeared at high concentrations (111 nM and 333 nM). Moreover, the time dependence of the effect of isradipine is shown intuitively in Supplementary Fig. S2b. In each concentration map, variation in feature parameters emerged within 15 min of treatment. However, the M1–M3 duration could not be extracted from the MB signals due to the inhibition of MB signals of isradipine at high concentrations of 111 nM and 333 nM. These results were also consistent with the statistical analysis and greatly facilitated visualized drug assessment by the EMS model.

The cardiomyocyte EMS biosensing system can achieve EMS signal recording in a high-throughput, high-content, and long-term manner. By defining and extracting the feature points of FP, MB, FP–MB signals, numerous new feature parameters were exploited for drug assessment. Compared with the conventional feature parameters, these new feature parameters provide an increased level of detail on electrical and mechanical signals and explore electrical-mechanical signal correlation as well. Based on these diverse feature parameters, the EMS system provided greatly improved drug assessment functionality, with high-content, dose-quantitative, and time-dependent performance.

## Conclusions and perspectives

In this study, we established a cardiomyocyte EMS biosensing system that enables high-content, dose-quantitative and time-dependent drug assessment. Multiple feature parameters can be extracted from FP, MB and FP–MB signals to examine dose-quantitative and time-dependent responses for drug screening. The feature parameters of the EMS model can promote rapid and effective identification of drug efficacy by heat map analysis. In this work, the EMS model demonstrates its potential as an alternative, quantitatively detailed approach to drug assessment. EMS biosensing in multiple channels can improve the accuracy of drug screening based on the relationship between various characteristic parameters of firing potential signals and mechanical beating signals and can reveal the time dependence of drug concentrations. This can significantly improve experimental efficiency and allow researchers to explore the correlation between drug concentration and FP–MB signals, which are highly favorable for revealing the in-depth influence of drugs on FP and MB signals. Despite these successes, however, the system has not achieved synchronized recording at a single-cell resolution. In future work, the coupling of single-cell technology with EMS is still needed to obtain higher-quality extracellular signals.

In the future, the EMS model can be further developed in the following aspects: (1) Combination with intracellular recording technology. The electrical signal quality of cardiomyocytes can be greatly improved by intracellular recording. (2) Development of single-cell analysis and recording technology. This technology would allow the accuracy and spatial resolution of the EMS model to be improved. (3) Integration with intracellular Ca^2+^ detection functionality. Excitation–contraction coupling can be investigated in depth by intracellular Ca^2+^ recording. With further improvement, the EMS biosensing system will have the potential to serve as a powerful tool for preclinical drug investigation.

## Supplementary information

Supplementary Figures

Conflict of interest
