# Supplementary material for: Cardiomyocyte electrical-mechanical synchronized model for high-content, dose-quantitative and time-dependent drug assessment
Source: Microsyst Nanoeng. 2021 Mar 25;7:26. doi: 10.1038/s41378-021-00247-0 (PMC8433219; doi:10.1038/s41378-021-00247-0)
Supplement: Supplementary file 1 — Supplementary Figures [file 41378_2021_247_MOESM1_ESM.docx]

**Supplementary**

**Cardiomyocyte electrical-mechanical synchronized model for high-content, dose-quantitative and time-dependent drug assessment**

***Jiaru Fang^1+^, Xinwei Wei^2+^, Hongbo Li^1+^, Ning Hu^1,3^*, Xingxing Liu^1^, Dongxin Xu^1^, Tao Zhang^1^, Hao Wan^2^, Ping Wang^2^*, Xi Xie^1^****

^1^ The First Affiliated Hospital of Sun Yat-Sen University; School of Electronics and Information Technology, Guangdong Province Key Laboratory of Display Material and Technology, Sun Yat-sen University, Guangzhou 510006, China.

^2^ Biosensor National Special Laboratory, Key Laboratory of Biomedical Engineering of Ministry of Education, Department of Biomedical Engineering, Zhejiang University, Hangzhou 310027, China.

^3^ State Key Laboratory of Transducer Technology, Chinese Academy of Sciences, Shanghai 200050, China.

^+^These authors contribute equally to this work.

*To whom correspondence may be addressed. Corresponding to: Xi Xie, xiexi27@mail.sysu.edu.cn; Ping Wang, cnpwang@zju.edu.cn; Ning Hu, [huning3@mail.sysu.edu.cn](mailto:huning3@mail.sysu.edu.cn);


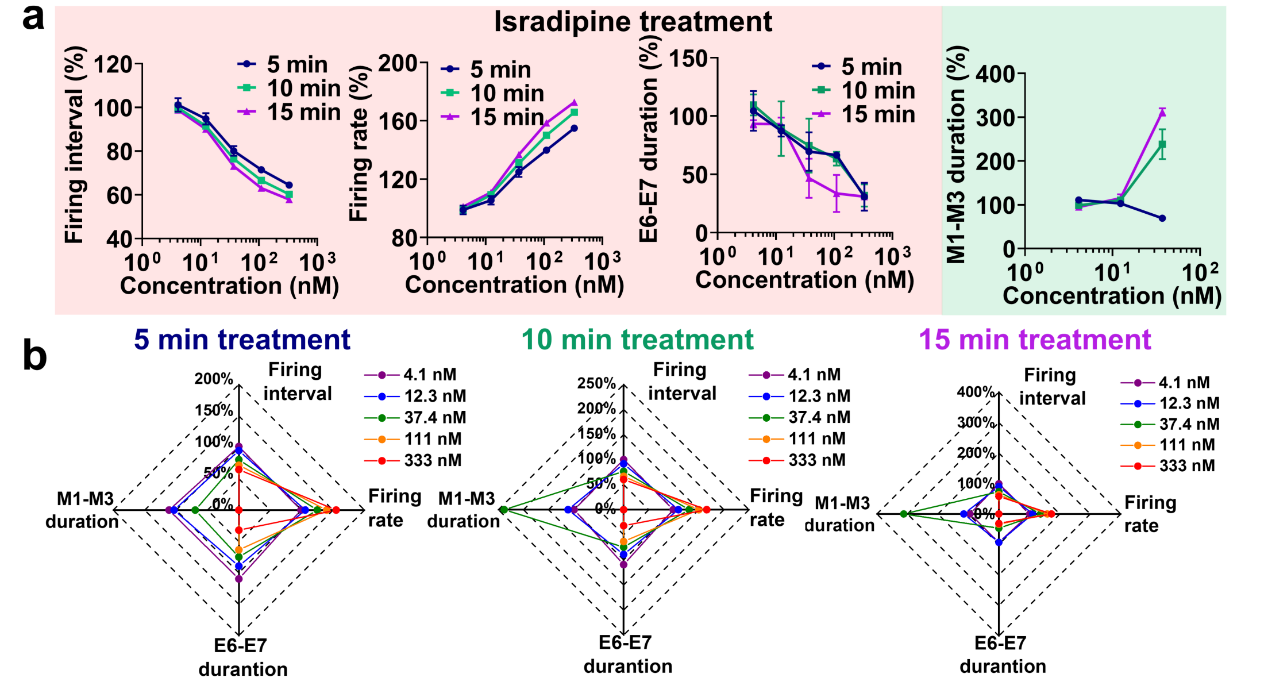


**Supplementary Figure S1.** Time-dependent assessment of isradipine by EMS model. (a) Statistical analysis of the time-dependent parameters under isradipine treatment including the firing interval, firing rate, E6-E7 duration and M1-M3 duration of extracellular electrical signals and mechanical beating singals. (b) Radar maps of all feature parameters analyzed in (a) at 5, 10, and 15 min drug treatment. In radar maps, the electrical signals of isradipine with different concentrations (4.1 nM, 12.3 nM, 37.4 nM, 111 nM, 333 nM) and the mechanical beating signals of isradipine with different concentrations (4.1 nM, 12.3 nM, 37.4 nM) displayed their specific patterns with given time of treatment.


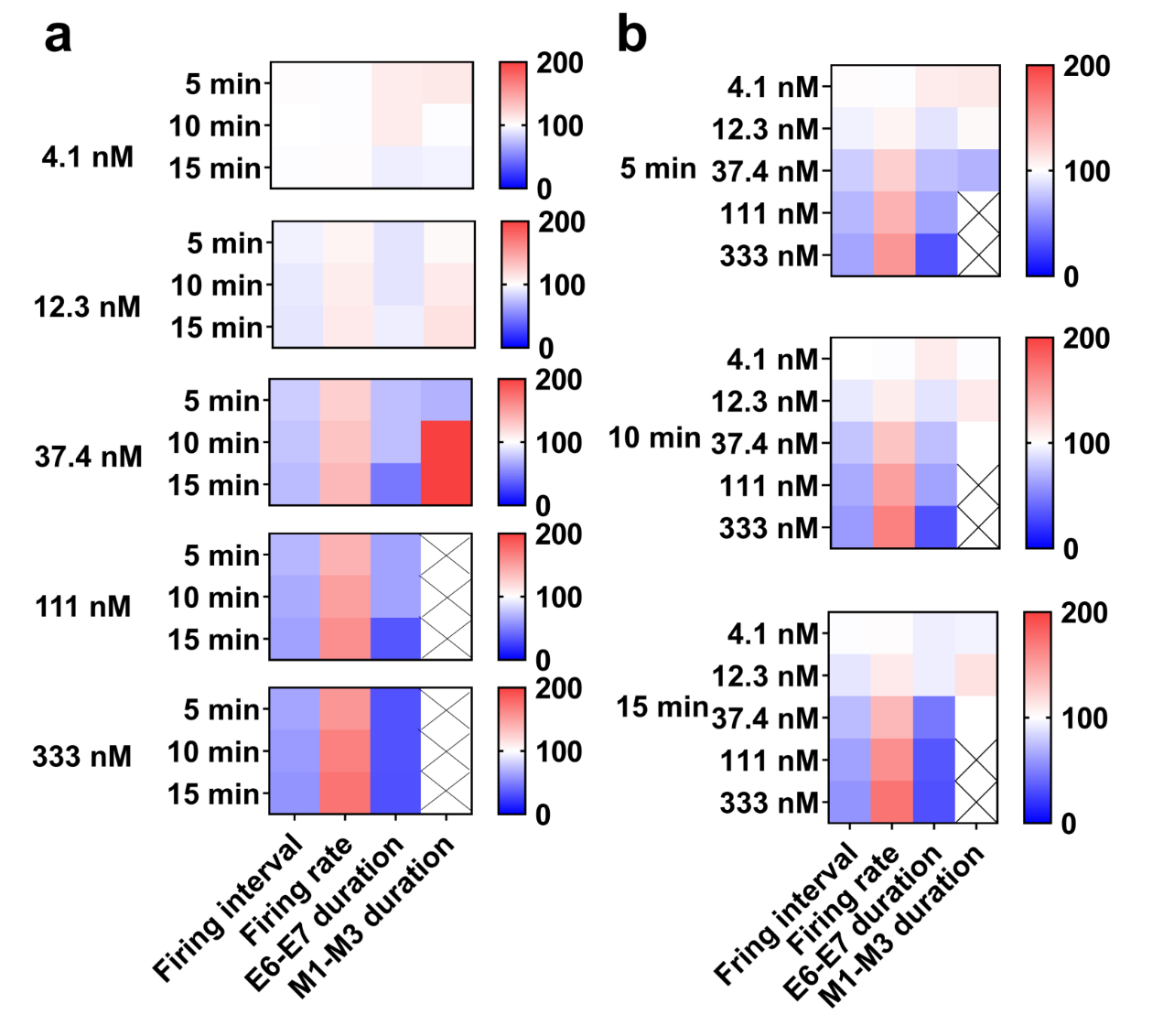


**Supplementary Figure S2**. Visualized analysis of EMS model under isradipine treatment by heat map. (a) Dose-quantitative specific patterns under isradipine treatment for 5, 10, and 15 min. Heat map with the concentrations ranging from 4.1 to 333 nM treatment at each time presented the similar patterns, indicating the similar variation of the feature parameters. (b) Time-dependent specific patterns within 15 min lidocaine treatment at the different doses. Heat map within 15 min treatment under each dose also presented the similar and obvious changes of feature parameters.


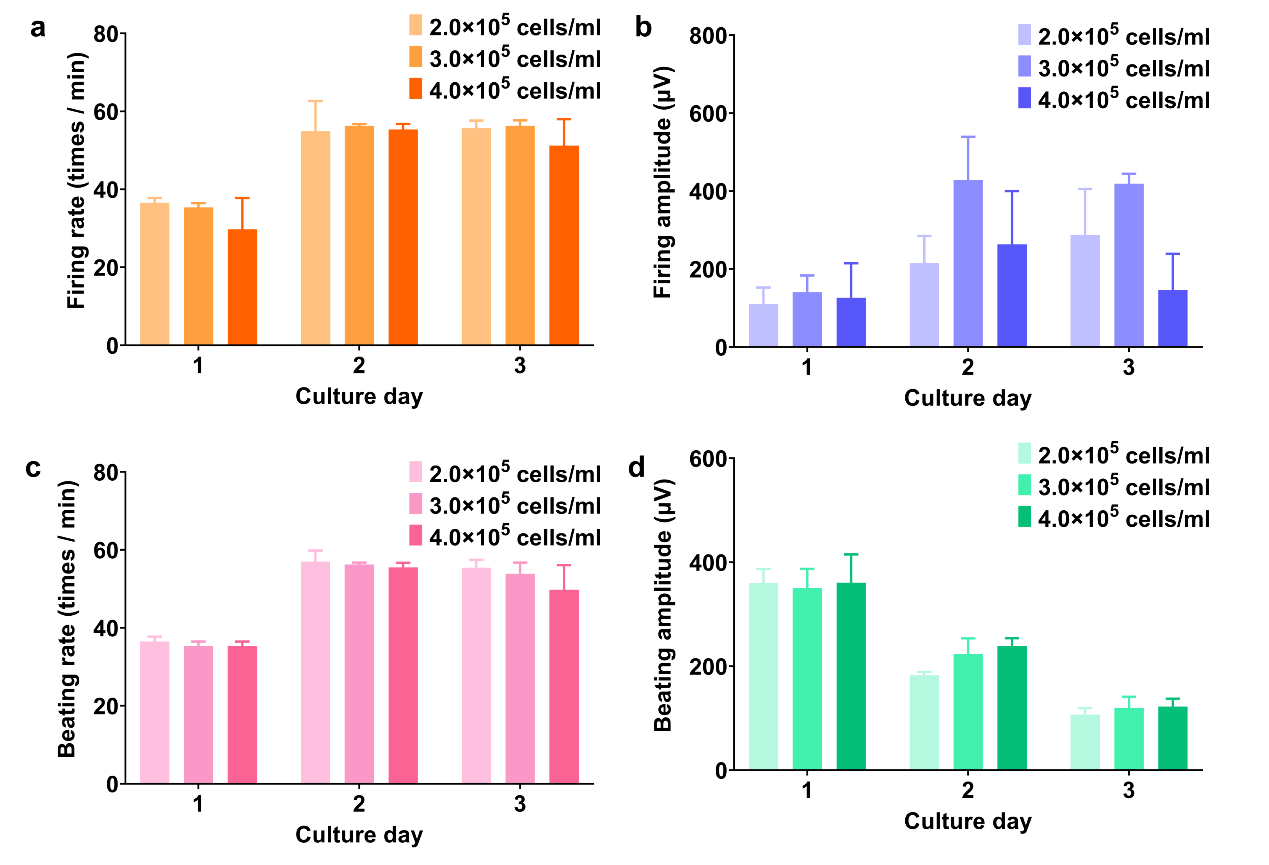


**Supplementary Figure S3.** Density optimization of cultured human iPSC (Human induced pluripotent stem cell-derived cardiomyocytes). (a) Statistical results of firing rate of different cell densities for 3-day culture. (b) Statistical results of firing amplitude of different cell densities for 3-day culture. (c) Statistic chart of beating rate of different cell densities for 3-day culture. (d) Statistic chart of beating amplitude of different cell densities for 3-day culture. (N=6)
